# Minimally invasive treatments for benign prostatic hyperplasia: A narrative review

**DOI:** 10.1097/MD.0000000000047951

**Published:** 2026-03-20

**Authors:** Xintao Zhang, Taisheng Liang, Yu Dong, Hongjun Gao

**Affiliations:** a Department of Graduate School, Guangxi University of Chinese Medicine, Nanning, Guangxi, China; b Department of Ruikang Hospital, Guangxi University of Chinese Medicine, Nanning, Guangxi, China.

**Keywords:** aquablation, benign prostatic hyperplasia, lower urinary tract symptoms, minimally invasive surgical therapies, prostatic artery embolization, prostatic urethral lift, temporary implantable device, water vapor thermal therapy

## Abstract

Benign prostatic hyperplasia-related lower urinary tract symptoms are highly prevalent in middle-aged and older men. Compared to traditional transurethral resection of the prostate (TURP), minimally invasive surgical therapies (MISTs) aim to provide meaningful symptom relief. This review evaluated the efficacy, safety, and procedural characteristics of 5 representative MISTs: water vapor thermal therapy (WVTT), prostatic urethral lift (PUL), prostatic artery embolization (PAE), temporary implantable nitinol device (iTIND), and Aquablation. A systematic literature search was conducted in PubMed (2018–2023), supplemented by backward citation tracking. Clinical studies reporting International prostate symptom score (IPSS), quality of life (QoL), and/or maximum flow rate (Qmax) were included, with higher-level evidence prioritized. Long-term data show that WVTT and PUL provide durable symptom improvement, achieving 48% IPSS reduction and 35% IPSS improvement, respectively, at 5 years. Aquablation has a lower rate of retrograde ejaculation than TURP. Importantly, no new sexual dysfunction has been reported after WVTT or PUL. The long-term retrograde ejaculation rate with iTIND is 4%. Most MISTs can be performed in an outpatient setting under local anesthesia. For prostates >80 cm^3^, PAE achieved >44% volume reduction. In summary, MISTs offer personalized treatment options for benign prostatic hyperplasia, though evidence strength varies. Aquablation has the strongest evidence for larger prostates, while WVTT and PUL are guideline-recommended for medium-sized glands. PAE is suitable for older/high-risk patients, and iTIND shows promise but requires more long-term data. Future research should focus on comparative trials to optimize patient selection.

## 1. Introduction

Benign prostatic hyperplasia (BPH) is caused by an increase in the number of smooth muscle and glandular epithelial cells in the transition zone of the prostate.^[1,2]^ Common urinary symptoms associated with BPH typically include frequency, urgency, difficulty urinating, interrupted flow, and increased nocturnal urination (nocturia).^[3]^ Less common symptoms include dysuria, reduced urine volume, and a sense of incomplete bladder emptying. The severity of these symptoms can be assessed using methods such as the International prostate symptom score (IPSS) and QoL measures.^[3–5]^

Although there are multiple causes for male lower urinary tract symptoms (LUTS), BPH is considered the primary cause of male LUTS. According to an international study, LUTS affects up to 60% of men worldwide, with an incidence rate of 18% in men over 40, 29% in men over 50, 40% in men over 60, and 56% in men over 70.^[6]^ Currently, the treatment options for BPH mainly include medication and surgical interventions. Surgical treatment should be considered when conservative and medical treatments are ineffective or when complications such as urinary retention, bladder stones, renal insufficiency, recurrent urinary tract infections, and gross hematuria arise as a result of BPH.^[7,8]^

While there have been no significant advancements in new drug treatments for BPH in recent years, minimally invasive surgical therapies (MISTs) have been developing rapidly over the past decade.^[9]^ The rapid progress of technology has led to the maturity of minimally invasive procedures for BPH, with TURP being one of the representative approaches. TURP is hailed as the gold standard in surgical treatment for LUTS in the history of BPH surgery, showing significant improvement in postoperative IPSS, QoL, and Qmax. Compared to traditional surgeries, TURP offers shorter recovery time and smaller surgical trauma. It is still recommended by major clinical guidelines as the standard treatment for moderate to severe LUTS.^[7,10,11]^

Although TURP is effective, it also has some limitations, such as increased intraoperative bleeding, urinary incontinence, urethral stricture, ejaculatory and erectile dysfunction (ED), and high retreatment rates. Studies report an incidence of urethral stricture ranging from 2.2% to 9.8% and a 5-year retreatment rate ranging from 3% to 14.5%.^[12]^ Despite its imperfections, tissue resection techniques provide better long-term improvements compared to non-resection techniques.^[13]^ For example, a recent study comparing TURP and prostatic artery embolization (PAE) after a 2-year follow-up demonstrated the superiority of TURP in terms of IPSS (difference of 2.88; *P* = .047) and QoL (difference of 0.99; *P* <.02) scores.^[14]^

Elderly patients prefer the use of local anesthesia to ensure higher safety during surgery, while younger patients request the preservation of erectile and ejaculatory functions. Patients also desire fewer postoperative complications and a more cost-effective approach. Unfortunately, TURP fails to meet these diverse patient demands adequately. Therefore, there is an urgent need for treatment options that offer improved safety, fewer complications, and better fulfillment of varied patient requirements.

Guided by the patient-centered approach to medical treatment, the fulfillment of diverse patient needs and addressing these treatment limitations requires the constant emergence of new MISTs. Compared to TURP, these MISTs offer multiple advantages such as less bleeding, faster recovery, greater cost-effectiveness, and lower incidence of complications. Most importantly, these procedures can be performed under local anesthesia in an office-based setting. However, the proliferation of these MISTs, coupled with the recent publication of pivotal long-term studies and updates to major guidelines, necessitates a contemporary evidence synthesis to guide clinical decision-making. In this review, we provide the latest technical advances and outcomes of 5 novel BPH techniques, including water vapor thermal therapy (WVTT), prostatic urethral lift (PUL), PAE, iTIND, and Aquablation. We focus on describing the principles, efficacy, complications, advantages over TURP, and the level of evidence for each technique.

## 2. Methods

This study reviews the efficacy of 5 MISTs for BPH: WVTT, PUL, PAE, iTIND, and Aquablation. The review was conducted by searching the PubMed database, focusing on literature from the past 5 years (2018–2023) to ensure timeliness, while also including key earlier studies through backward citation searching. Search terms included keywords such as “BPH,” “minimally invasive surgical therapy,” and the names of each technique. Inclusion criteria included clinical studies reporting outcomes such as IPSS, QoL, and Qmax; non-English literature, case reports, and small sample studies (n <20) were excluded, as studies with small sample sizes may lead to insufficient statistical power, affecting the reliability of the results. In terms of evidence selection, priority was given to high-level evidence such as randomized controlled trials and multicenter studies.

## 3. Results

### 3.1. Comparison of clinical characteristics between MISTs and TURP

As shown in Table 1, this review compares the main differences between 5 MISTs technologies and traditional TURP in terms of anesthesia suitability, suitability for large prostate volumes, surgical efficiency, functional preservation, and treatment durability. Specifically, regarding anesthesia, WVTT, PUL, PAE, and iTIND can all be performed under local anesthesia, while Aquablation requires general anesthesia. For patients with large prostate volumes, PAE, iTIND, and Aquablation show good suitability, whereas WVTT and PUL are not suitable. In terms of surgical efficiency, WVTT has the shortest operative time (5–10 minutes), while PAE is the longest (50–150 minutes); the hospital stay for all MISTs technologies is significantly shorter than that for TURP, with Aquablation having an average hospital stay of 1.4 days, and the other technologies marked as “short.”

**Table 1:** Advantages and disadvantages of minimally invasive surgical therapies compared to TURP.

Surgery name	Rezum	PUL	PAE	iTIND	Aquablation
SULA	Yes^[15]^	Yes^[16]^	Yes^[17,18]^	Yes^[19]^	No^[20]^
LPF	No^[7,21]^	No^[7,22]^	Yes^[23]^	Yes^[24]^	Yes^[25,26]^
OP (mins)	5–10^[16]^	30–45^[22]^	50–150^[27,28]^	15–30^[29]^	33^[30]^
LOS	Short^[15]^	Short^[16]^	Short^[17,18]^	Short^[19]^	1.4days^[20,31]^
RER	0%^[32]^	0%^[33]^	6%^[14]^	0%^[19,34]^	10%^[20]^
RR	4.4%^[21,32]^	13.6%^[33,35]^	21.1%^[36]^	4%^[37]^	4%^[20]^

iTIND = temporary implantable device, LOS = length of stay, LPF = large prostate function, MISTs = minimally invasive surgical therapies, OP (mins) = operation time (minutes), PAE = prostatic artery embolization, PUL = prostatic urethral lift, RER = retrograde ejaculation rate, RR = retreatment rate, SULA = surgery under local anesthesia, TURP = transurethral resection of the prostate.

### 3.2. Summary of the clinical positioning and levels of evidence of 5 novel MISTs

Table 2 summarizes the clinical positioning and levels of evidence for the 5 novel MISTs. It details the core advantages, current evidence base with guideline recommendations from the American Urological Association (AUA) and European Association of Urology (EAU), and the ideal patient profile for each procedure. Notably, WVTT and PUL are supported by 5-year RCT data and are highlighted for their excellent preservation of ejaculatory and sexual function. Aquablation is also supported by 5-year RCT data, demonstrating non-inferior efficacy to TURP with preserved sexual function, particularly suitable for larger glands. In contrast, while PAE offers a completely incision-free approach for high-risk patients or very large prostates, its long-term efficacy requires further verification, and guideline recommendations are cautious. iTIND shows promising short-term outcomes but lacks long-term comparative data and is not yet formally recommended in major guidelines.

**Table 2:** Summary of the clinical positioning and levels of evidence of 5 novel MISTs.

Surgery name	Core advantages/features	Current evidence and guideline status	Ideal patient profile
Rezum	Steam thermotherapy, preserves ejaculation function, outpatient, local anesthesia.	Robust evidence:5 yr of RCT data.^[21,32]^ Guideline recommendation: moderate recommendation by AUA (for prostate volumes of 30–80 cm^3^)^[7]^; not included in EAU.	Patients who wish to preserve ejaculation function and have a moderate prostate volume (30–80 cm^3^).
PUL	Mechanical dilation of the urethra, no tissue removal, excellent preservation of sexual function.	Robust evidence:5 yr of RCT data.^[33]^ Guideline Recommendation: both AUA and EAU recommend (for patients with no median lobe obstruction and prostate volume <80 cm^3^).^[7,22]^	The strong requirement is to preserve sexual function, with the primary focus on lateral lobe hyperplasia and moderate glandular size.
PAE	Completely incision-free, endovascular intervention, suitable for large prostates (informed choice).^[22]^	Robust evidence:Long-term efficacy needs to be verified.^[17,18,36,38]^ Guideline Recommendation: AUA does not recommend and high-risk patients.	Patients with high-surgical risk, those who refuse invasive surgery, or those with very large glands (>80 cm^3^). routine use^[7]^; EAU gives a weak recommendation
iTIND	Temporary implant (5–7 d), works by remodeling, preserves sexual function, quick recovery.	Robust evidence:Short-term effective and safe,^[19,24,34]^ but lacks long-term comparative data. Guideline Recommendation:Not yet officially recommended by AUA/EAU guidelines.	Desires minimally invasive surgery to preserve sexual function and accepts temporary implantation.
Aquablation	Robot-assisted water jet ablation, precise for large prostates, short learningcurve.	Robust evidence:5-yr RCT data versus TURP, confirming non-inferiority and preservation of sexual effective function.^[20,39]^ Guideline recommendation: AUA (conditional)/EAU (weak) recommend as an alternative to TURP.^[7,22]^	Seeking effective treatment that preserves sexual function despite large gland size (>50 cm^3^).

iTIND = temporary implantable device, MISTs = minimally invasive surgical therapies, PAE = prostatic artery embolization, TURP = transurethral resection of the prostate.

## 4. Rezum

**Figure 1.**
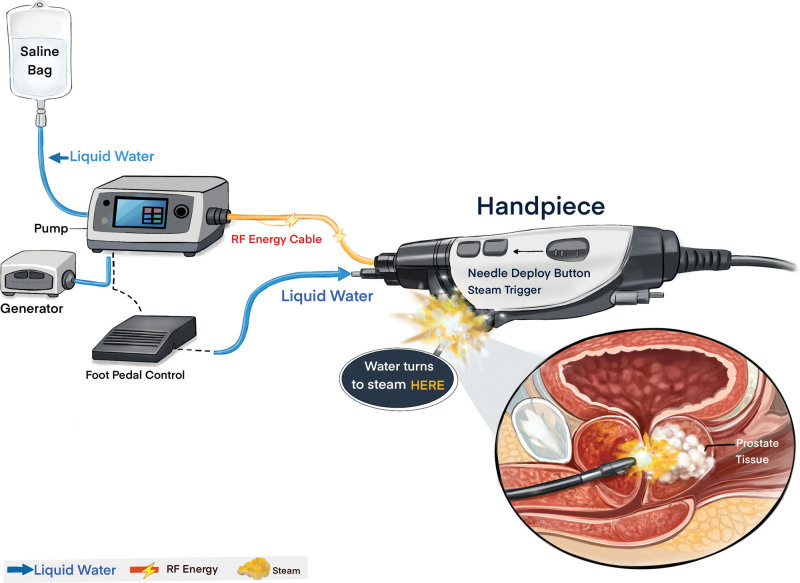
Schematic diagram illustrating the convective water vapor ablation mechanism of Rezum therapy. This schematic, adapted and annotated from Boston Scientific materials, visually elucidates the core therapeutic principle described in the manuscript.The process begins with the conversion of sterile water into water vapor within the Generator (corresponding to the text: “converts sterile water into steam”). The vapor is then delivered, controlled, and released via the delivery device, which is equipped with a retractable shaft and needle tip (corresponding to the text: “introduces steam energy… using a specific cystoscope with a puncture needle”). The core mechanism of action is conceptually illustrated: vapor injected through the needle tip rapidly disperses and transfers thermal energy via convection within the prostate tissue (corresponding to the text: “achieves heat transfer through convection”), leading to irreversible necrosis of target cells. It is this highly confined thermal effect that ensures ablation is restricted to the target hyperplastic tissue, thereby underpinning the clinically reported preservation of peri-prostatic anatomical structures, such as the urethra and tissues relevant to sexual function, as discussed in the text.

As the predominant commercial embodiment of WVTT, Rezum (Boston Scientific Corporation, Marlborough) uses radiofrequency energy to convert sterile water into steam and uses the thermal energy of steam to ablate prostate tissue. Rezum achieves heat transfer through convection and introduces steam energy into the anatomical structure of the prostate using a specific cystoscope with a puncture needle. The release of thermal energy leads to irreversible cell membrane damage and cell death. Compared with transurethral microwave therapy and transurethral needle ablation, the necrotic tissue will decrease in temperature in a shorter time, and the resulting cell debris will be absorbed by the body after surgery^[40]^ (Fig. 1). Throughout the entire surgical process, there is no obvious thermal effect outside the prostate tissue range, so the urethra, bladder neck, and external sphincter are not affected.^[15]^

Rezum was approved by the US FDA in 2015, and many Rezum studies have been published to date. Mcvary et al^[21]^ published a prospective, multicenter, double-blind, randomized controlled trial in 2019, which included 135 men who received Rezum treatment and 61 men who underwent sham cystoscopy. The treatment group and the sham control group were in a ratio of 2:1, and the follow-up time after surgery was 4 years. The inclusion criteria were prostate volume between 30 and 80 cm^3^ without morphological restrictions. The study confirmed that within 3 months after surgery, the IPSS of the treatment group improved by 47%, QoL improved by 43%, and Qmax increased by 50%, and the effect could last for 4 years after surgery. The rate of surgical retreatment was 4.4%, and there was no damage to sexual function. Mcvary et al^[32]^ released the final 5-year results in 2021, which is the highest level of evidence supporting the safety and efficacy of Rezum so far. The final study data showed continuous improvement over 5 years, with IPSS decreasing by 48% compared to baseline, QoL decreasing by 45% compared to baseline, and Qmax increasing by 49%. The medical retreatment rate of the treatment group patients was 11.1%, and the rate of surgical retreatment remained at 4.4%.^[32]^ No cases of ED or RR were found in the treatment group patients.^[32]^ Most adverse events of Rezum are transient, mild (Clavien I–II), and easy to resolve. However, Mollengarden et al^[41]^ observed complications of Clavien III/IV in postoperative patients. The most common adverse events were postoperative urinary retention (3.7%), urinary frequency or urgency (5.9%), hematuria (11.8%), difficulty urinating (16.9%), and urinary tract infection (3.7%).^[32]^ It should be noted that these studies were all sham-controlled trials, with the risk of unexpected unblinding, which may reduce the impact of the placebo group and exaggerate the effect of the treatment group.^[10]^ Furthermore, while the cited 5-year data provide robust evidence for the overall cohort, long-term outcomes specifically in certain subgroups, such as patients with very large prostates (>80 cm^3^) or those with predominant median lobe enlargement, remain less well-characterized and warrant further investigation.

According to the AUA 2021 guidelines, for patients with BPH and prostate volume between 30 and 80 cm^3^, the Rezum system should be considered as a treatment option (moderate recommendation, level C evidence) for those who wish to preserve ejaculation and erectile function (conditional recommendation, level C evidence).^[7]^ However, the EAU guidelines did not make a clear statement on the Rezum system and did not include it as a recommended treatment method.^[22]^

## 5. Prostatic urethral lift

**Figure 2.**
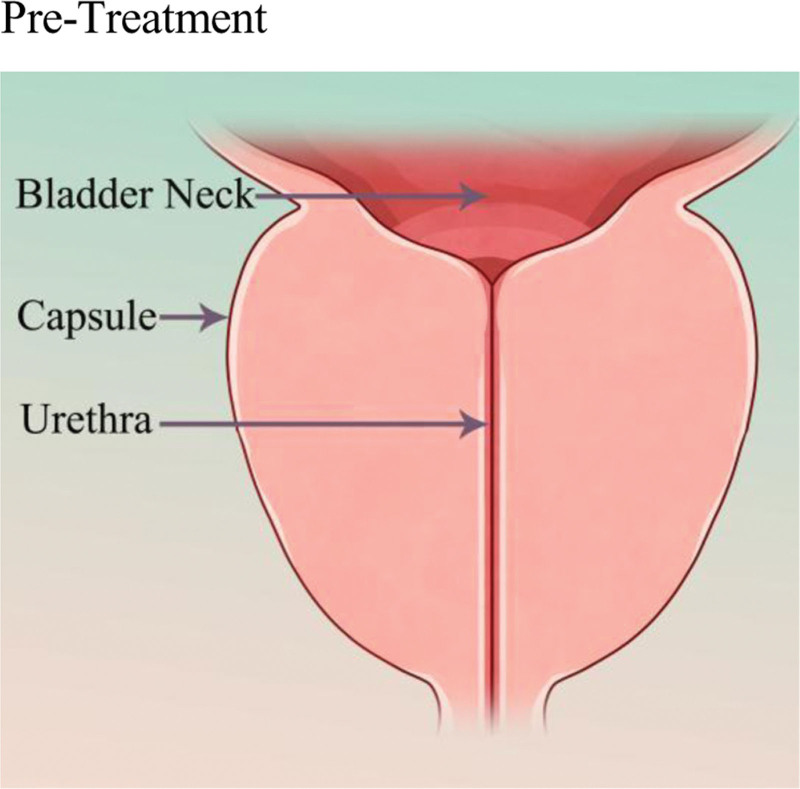
Normal anatomy of the bladder outlet and prostatic urethra. This schematic cross-section clearly illustrates the normal anatomical relationships between the bladder neck, prostatic capsule, and the urethra traversing through it. This figure provides a visual baseline for understanding the pathological state of urethral compression and narrowing caused by BPH, corresponding to the anatomical basis of BPH-induced lower LUTS described in the text. BPH = benign prostatic hyperplasia, LUTS = lower urinary tract symptoms.

**Figure 3.**
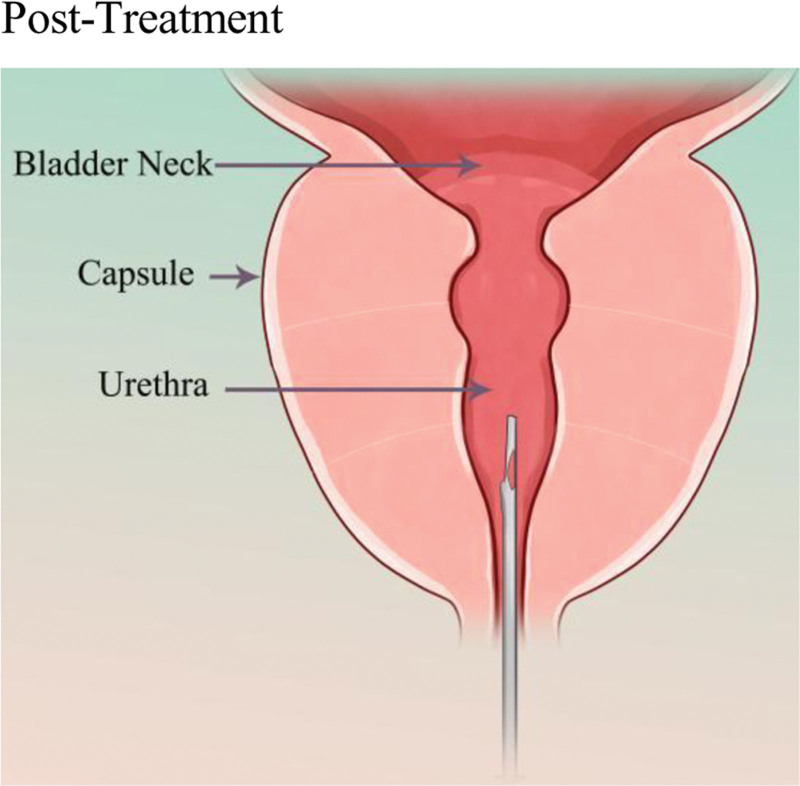
Schematic diagram of the PUL mechanism. This figure visually demonstrates the core procedure of the PUL surgery: under cystoscopic guidance, an implant (represented by the needle-like device in the figure) penetrates the urethral wall. After its distal end (intraurethral) and proximal end (extra-capsular) are anchored, it generates a continuous lateral retracting force (as indicated by the arrows). This mechanical pulling action enlarges the urethral lumen without tissue resection, vividly illustrating the principle of “altering the anatomy to establish a continuous channel” as described in the text. This mechanism is directly linked to the key clinical feature that distinguishes PUL from TURP: it avoids thermal damage to the peri-verumontanum tissues. This provides an intuitive anatomical and physiological explanation for the “superior ejaculatory function preservation rate” demonstrated by PUL in multiple studies.^[33,42]^ PUL = prostatic urethral lift, TURP = transurethral resection of the prostate.

The basic principle of UroLift (Neotract Inc., Pleasanton) or PUL is to separate and pull the enlarged prostate tissue by permanently implanting devices in order to establish a continuous channel in the prostatic urethral portion. The specific procedure involves the doctor guiding small permanent suture implants into place under cystoscopy, with 1 end fixed in the urethra and the other end fixed on the outer surface of the prostate capsule (Figs. 2 and 3). This changes the anatomical structure of the prostate without needing to cut or remove the enlarged prostate tissue, thus enlarging the narrowed urethra and relieving symptoms.^[43]^ The number of implants required depends on the presence of the median lobe, the length of the prostatic urethra, and the size of the prostate. The surgery usually takes <1 hour and can be performed under local or general anesthesia in an outpatient setting.

A study by Gratzke et al^[16]^ directly compared PUL with TURP, with a follow-up time of 24 months. The TURP group had a significant advantage in terms of IPPS and Qmax, but the PUL group performed better in preserving ejaculation function, residual urine volume after emptying, and recovery speed. However, a meta-analysis by Franco et al^[13]^ showed that there may be little difference in IPSS between PUL and TURP after surgery. The 2-year surgical retreatment rate was 5.7% in the TURP group and 13.6% in the PUL group.^[16]^

In a multicenter study published in 2017, PUL was compared with a sham treatment and followed up for 5 years.^[33]^ The results showed that the average improvement in IPSS was 35%, Qol improved by 50%, and Qmax increased by 50% in the treatment group. The improvement persisted over the 5-year period. No reports of new-onset ejaculation dysfunction or ED were reported, and the 5-year surgical retreatment rate was 13.6%,^[33]^ consistent with the conclusion of Gratzke et al 2-year study.^[35]^ Multiple studies have shown that patients undergoing PUL have a retreatment rate of 8% to 20%, and the retreatment rate of PUL (8%) seems to be higher than that of TURP (6%), but PUL performs better in preserving ejaculation function.^[16,33,35,44]^ Most complications are mild in nature (Clavien grade 1) and temporary, including urinary retention, urinary tract irritation symptoms, difficulty urinating, and bleeding. There are also a few cases of prostatitis, urinary tract infection, and epididymitis, but most symptoms disappear within 2 to 4 weeks after surgery.^[27,28,33,42,45,46]^ A meta-analysis published in 2023 suggests that PUL seems to be advantageous from a risk-benefit perspective, although it may be inferior to TURP in objective outcomes, and these findings are worth confirming through long-term randomized controlled trials^[47]^

The AUA 2021 guidelines recommend PUL as a treatment option (moderate recommendation, level C evidence) for LUTS/BPH patients, especially for those with prostate volume of 30 to 80 cm^3^ and confirmed absence of obstructive median lobe BPH. PUL can also be considered as a treatment choice (conditional recommendation, level C evidence) for patients who wish to preserve erectile and ejaculatory function.^[7]^ The EAU 2023 guidelines suggest that patients with BPH and prostate volume <70 cm^3^ and without obstructive median lobe can choose PUL (strong recommendation), but it should be noted that PUL has not yet been validated by long-term clinical studies.^[22]^

Although the above guidelines do not include BPH patients with obstructive median lobe in the recommended range, the MedLift study published in 2018 showed that BPH patients with obstructive median lobe who received PUL treatment had similar improvements in IPSS, QoL, and Qmax as those with only unilateral enlargement during a 1-year follow-up. Approximately 40% of patients showed improved erectile function, and the retreatment rate was 2%. This suggests that PUL may also be effective for BPH patients with obstructive median lobe.^[48]^

When interpreting the evidence above, several limitations should be considered. Key randomized controlled trials supporting the efficacy of PUL (e.g.,^[33]^) employed a sham‑controlled design; however, due to the presence of implants, maintaining complete blinding is challenging, which may influence the assessment of subjective outcome measures such as the IPSS. Moreover, although 5-year follow‑up data are available, long‑term (>5-year) efficacy, retreatment rates, and implant durability in specific subgroups – such as patients with very large prostates (>80 cm^3^), prominent median lobe enlargement, or coexisting significant bladder dysfunction – remain inadequately characterized and require further validation through longer‑term prospective studies.

## 6. Prostate artery embolization

**Figure 4.**
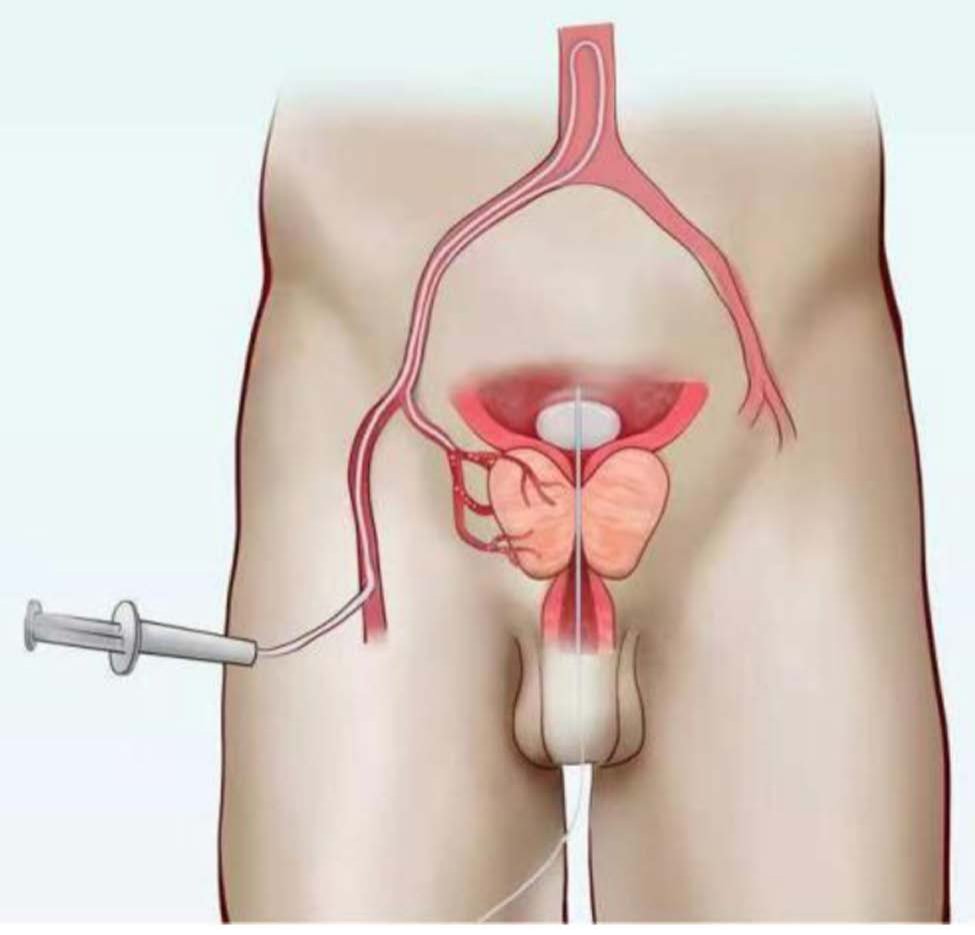
Schematic diagram of the endovascular approach for PAE. Narrative context and integration with arguments: this figure visually presents the anatomical basis of PAE as a minimally invasive outpatient procedure. The depicted single percutaneous femoral artery puncture access provides a technical-pathway explanation for how this procedure can be performed under local anesthesia, potentially avoiding the risks and costs associated with general anesthesia and hospitalization. This visual representation helps illustrate the minimally invasive and convenient operational characteristics of PAE, offering tangible support for discussions regarding its cost control (as mentioned in the text, e.g., “approximately one-third the cost of TURP”) and its suitability for specific patient populations (e.g., those with higher surgical risk). PAE = prostatic artery embolization, TURP = transurethral resection of the prostate.

**Figure 5.**
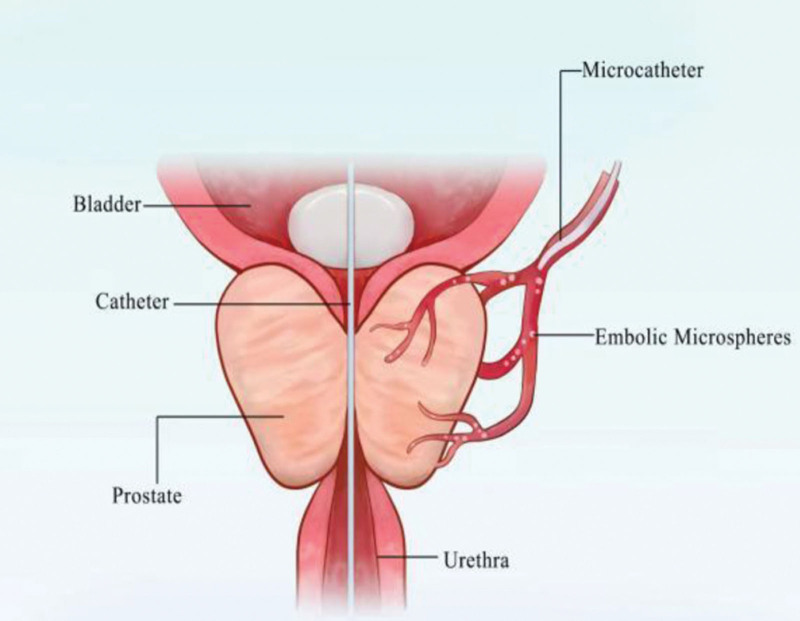
Schematic diagram of the principle of super-selective PAE. Narrative Context and Integration with Arguments: This figure details the core pathophysiological process through which PAE exerts its therapeutic effect. The illustration of a microcatheter super-selectively entering the prostatic feeding artery and releasing embolic microspheres visualizes the key step described in the text: “occluding the arteries, thus blocking its blood supply.” The resulting targeted ischemia is the initiating mechanism that subsequently leads to prostatic tissue atrophy and volume reduction (with literature reporting an average reduction of >44%), which may consequently alleviate urethral compression and relieve LUTS. This figure, therefore, aids the reader in understanding the logical sequence from the interventional procedure to the clinical outcomes, serving as a crucial visual reference for evaluating the efficacy of PAE. LUTS = lower urinary tract symptoms, PAE = prostatic artery embolization.

The fundamental mechanism of PAE is to induce ischemia or hypoxia in prostate tissue, thereby leading to cell necrosis, apoptosis, fibrosis, and reduction of blood supply to the hyperplastic gland through prostate contraction. This process is accompanied by partial or complete glandular cystic transformation, ultimately alleviating urinary difficulties in patients.^[49,50]^ Prior to the procedure, computed tomography or magnetic resonance angiography is typically performed to evaluate the anatomical structure of the pelvic arteries and digital subtraction angiography of the internal iliac artery is used to assess prostate blood supply.^[51]^ Subsequently, superselective microcatheterization and embolization of the prostatic arteries are performed, with the injection of specific embolic agents through the catheter to occlude the arteries supplying the prostate, thus blocking its blood supply (Figs. 4 and 5).

PAE was previously limited to the treatment of significant hematuria until DeMeritt et al^[49]^ reported a case in 2000 where polyvinyl alcohol particles were used for PAE to treat hematuria caused by BPH. Immediately after the cessation of hematuria, the patient reported an improvement in BPH symptoms. The researchers also observed a reduction in prostate volume by 52% and 62% at the 5-month and 12-month follow-ups, respectively, compared to the initial volume.^[49]^ This demonstrates that PAE can decrease both the volume and blood supply of the prostate, making it a potential adjunct therapy for the management of refractory hematuria associated with BPH.^[52]^

PAE is not as effective as TURP in improving BPH symptoms and urodynamic parameters.^[14,53,54]^ Recent meta-analyses have also shown that TURP has better outcomes for the majority of patients compared to PAE.^[55,56]^ A meta-analysis published in 2023 confirmed that PAE may have poorer short-term efficacy on urine flow but acknowledged its position as an alternative to ablative surgeries, with data indicating that it can achieve improvements in IPSS, QoL, Qmax, and other aspects that are comparable to those of TURP in many patients.^[47]^ The reason for the inferior short-term efficacy compared to TURP is that the pathological changes in prostate tissue after a reduction in blood supply are not immediate and require more time to achieve optimal results. Nevertheless, multiple studies have demonstrated a reduction in prostate volume by over 44% after PAE treatment, effectively alleviating BPH-induced LUTS. However, there is a postoperative retreatment rate of 20% to 23%.^[17,18,36,38]^ Additionally, Wang et al^[57]^ found that PAE was more effective in treating BPH patients with larger prostate volumes (>80 cm^3^) compared to those with moderate-sized prostates (50–80 cm^3^), and larger prostate volume did not pose additional risks to patients.^[57]^

While PAE can be considered as one of the treatment options for prostate enlargement, current data does not support the routine use of PAE for treating LUTS/BPH, and the balance of benefits and risks remains unclear. Therefore, it is not recommended to use PAE outside of clinical trials.^[7]^ However, the EAU 2023 guidelines suggest that PAE can be offered to men with moderate to severe LUTS, provided they consider minimally invasive treatment options and are willing to accept outcomes that may not be as favorable as TURP (weak recommendation).^[22]^ In summary, evidence from multiple studies indicates that PAE leads to improvement across key objective outcome measures in patients with moderate to severe LUTS due to BPH.^[29,58]^ Therefore, it can be considered a treatment option for BPH, particularly in patients who refuse or cannot tolerate surgery. In recent years, PAE has gained recognition among patients worldwide, possibly due to its safety, cost-effectiveness, and convenience. PAE can be performed under local anesthesia in an office setting, and it eliminates the costs associated with hospitalization and anesthesia, only accounting for one-third of the cost of TURP.^[13,59,60]^ PAE has now become a widely accepted minimally invasive procedure by several radiology societies for the treatment of LUTS caused by BPH, although it is not considered a primary treatment option by the AUA.^[7,23]^

## 7. iTIND

**Figure 6.**
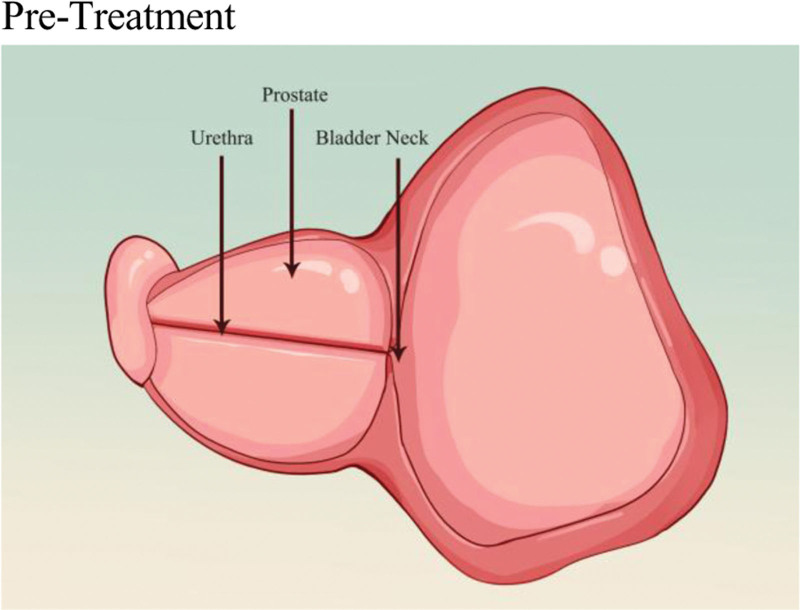
This panel illustrates the anatomical context for iTIND placement, showing the prostate, prostatic urethra, and bladder neck. The device is implanted via the urethra using a cystoscope, an approach that does not require a skin incision. This purely endoluminal technique is compatible with clinical practices typically employing only intravenous sedation or local anesthesia and may facilitate same-day patient discharge. The schematic depicts the conceptual spatial configuration of the device, with its anchoring element positioned at the bladder neck and the struts within the prostatic urethra. iTIND = temporary implantable device.

**Figure 7.**
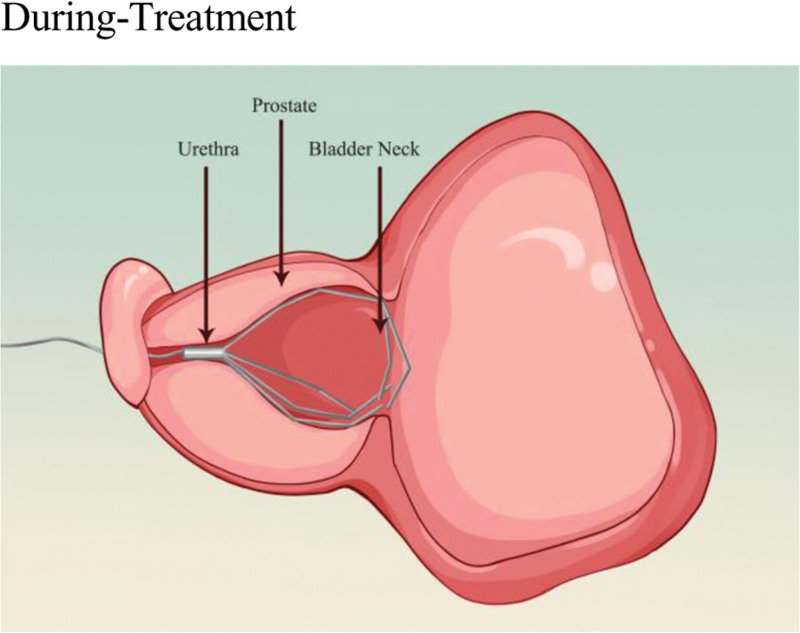
This cross-sectional view details the device’s mechanism. Three nitinol struts are shown positioned at approximately the 5, 7, and 12 o’clock positions within the prostatic urethra. Arrows indicate that these struts are designed to exert continuous, localized radial compressive force on the surrounding prostatic tissue. This inward mechanical compression is believed to be the key process that initiates localized ischemic changes, leading to subsequent tissue healing and remodeling. In contrast to technologies based on thermal ablation or resection, this physical mechanism theoretically avoids thermal damage or direct tissue removal. This may underlie the potential for preserving erectile and ejaculatory function, as reported in several clinical studies (e.g., data showing no worsening or even improvement in IIEF scores). IIEF = International Index of Erectile Function.

**Figure 8.**
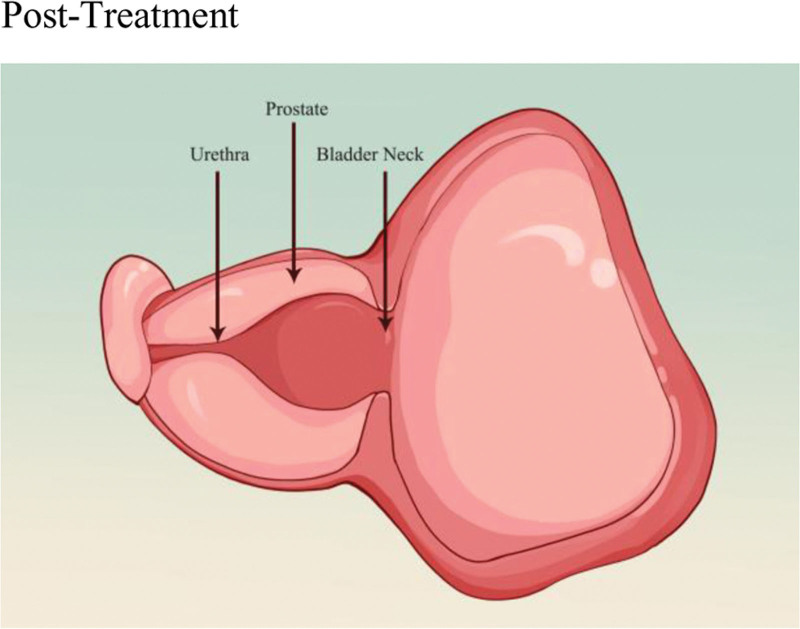
Following a 5 to 7 d dwell time and subsequent device removal, the compressed prostatic urethral tissue remodels to form a relatively wider lumen. This intended structural modification provides the anatomical basis for the improvements in Qmax and IPSS observed in clinical studies of iTIND. IPSS = International prostate symptom score, iTIND = temporary implantable device.

The second-generation iTIND system (Medi-Tate, Hadera, Israel) is a temporary implant made of nitinol and is placed in the bladder outlet, prostatic urethra, and bladder neck.^[24,61,62]^ The device consists of 3 nitinol alloy struts connected at the distal end, an anti-migration anchoring leaflet, and a polyester retrieval suture.^[62]^ Surgeons use a rigid cystoscope via the transurethral route to pass the closed device through a cystoscope sheath into the filled bladder. The device is then deployed and retracted, positioning the anchoring leaflet at the 6 o’clock position below the bladder neck, and the 3 struts at the 5 o’clock, 7 o’clock, and 12 o’clock positions inside the prostatic urethra.^[62,63]^ The 3 struts apply continuous pressure on the prostatic urethra, leading to localized ischemic necrosis and remodeling of the bladder neck and prostatic urethra, thereby reducing bladder outlet obstruction and relieving LUTS^[24,62,63]^ (Figs. 6–8).

De Nunzio et al^[64]^ reported the 6-month results of a prospective, multicenter study involving 70 male patients who underwent iTIND treatment. The inclusion criteria were expanded to include patients with a prostate volume <120 cm^3^. IPSS, QoL, and Qmax showed significant improvements compared to baseline levels (*P* <.0001), with reductions in IPSS (21.2–8.3), QoL (4.1–2.0), and increases in Qmax (7.3–12.0).^[64]^ In a 36-month follow-up of the same patient cohort, continuous improvements in IPSS and Qmax were observed compared to baseline data, with a median QoL score of approximately 2.^[65]^ Porpiglia et al^[24]^ also conducted a single-arm, multicenter, international prospective study involving 81 patients who underwent iTIND treatment and were followed up for 1 year. Significant improvements were observed in IPSS, QoL, and Qmax, with no reports of sexual or ejaculatory dysfunction, and patients were discharged on the day of surgery.^[24]^

It is worth noting that a multicenter, randomized controlled, prospective trial by Chughtai et al^[19]^ on iTIND showed that at 3 months post-surgery, 78.6% of the iTIND patients experienced a decrease of 3 points in IPSS compared to 60% of the sham patients. At 12 months post-surgery, the iTIND group showed improvements in IPSS (−9.25), QoL (−1.9), and Qmax (+3.5). In the iTIND group and control group, 38.1% and 17.5% of patients, respectively, experienced transient minor adverse events, mostly classified as Clavien-Dindo Grade I or II. The rate of Grade III adverse events was only 9.9%, primarily consisting of acute urinary retention, with no reported incidents of new-onset RR or ED.

Compared to TURP, iTIND may have a higher incidence of acute urinary retention, and among the 5 BPH new technologies, iTIND has the lowest likelihood of improving IPSS but may result in a significant reduction in major adverse events.^[13,37]^

While longer-term data are still evolving, a 2023 single-arm study provided preliminary outcomes at >48 months for iTIND.^[37]^ In this study, durable improvements in IPSS and QoL were reported, with no late postoperative complications noted after 36 months and a 4% retreatment rate. Nevertheless, the long-term evidence base remains limited to such uncontrolled cohorts, underscoring the need for further comparative studies with extended follow-up.

A multicenter, randomized, single-blinded, placebo-controlled trial studied 185 men with BPH with an average age of 61.1 ± 6.5 years.^[34]^ They were randomly assigned in a 2:1 ratio to the iTIND group and the sham surgery group. The iTIND device was left in place for 5 to 7 days, and an 18F Foley catheter was inserted and removed in both the iTIND and sham surgery groups. Patients were evaluated using the International Index of Erectile Function at baseline, 3 months, and 12 months. In the iTIND group, men without ED at baseline showed an improvement of + 6.07 ± 21.17 points in the International Index of Erectile Function total score at 12 months (*P* = .034), and there was also improvement in ejaculatory function. Regardless of age, prostate volume, or baseline sexual function, no changes in sexual function or ejaculatory function were observed in the iTIND group, indicating a protective effect of iTIND on sexual and ejaculatory function.

iTIND not only demonstrates precise short-term efficacy and shorter surgical time but also allows for same-day discharge. Among the emerging technologies used in the treatment of BPH, iTIND is a promising option for those who wish to preserve sexual function and desire good tolerability. Although TURP may be more effective, these advantages are particularly appealing for certain patient populations.^[66]^ iTIND can be performed under intravenous sedation or local anesthesia alone, making it suitable for elderly and high-risk BPH patients.

While iTIND presents a minimally invasive option with promising functional preservation, its long-term durability remains to be fully established beyond the available single-arm studies. Consequently, and importantly, it has not yet met the evidence threshold for inclusion in the major AUA and EAU guidelines,^[7,22]^ which is a key consideration for its current clinical adoption.

## 8. Aquablation

**Figure 9.**
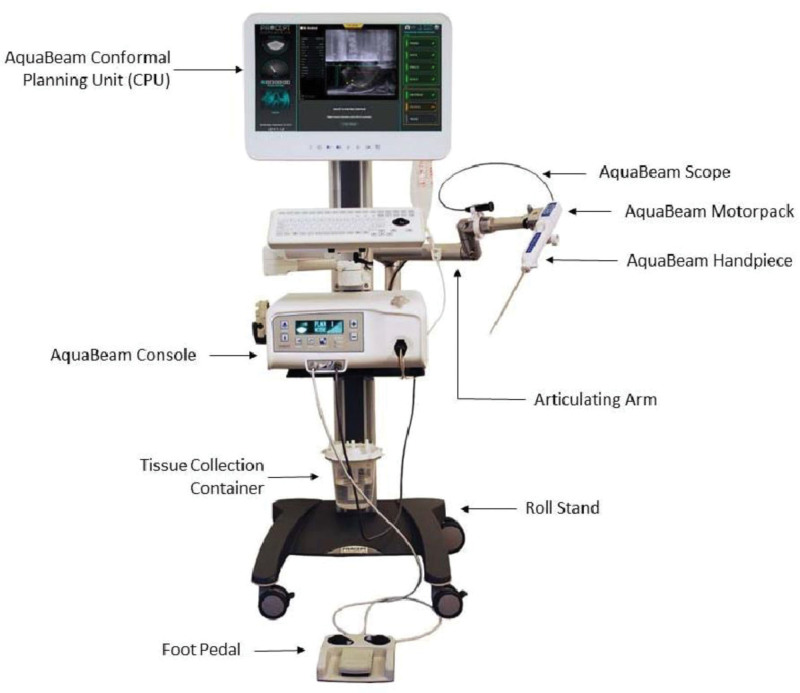
AQUABEAM system. (From public open access FDA approval https://www.accessdata.fda.gov/cdrh_docs/ reviews/DEN170024.pdf.) This figure illustrates the corecomponents of the AquaBeam system, whose integrated operation visually explains the key technical principles underlying the clinical outcomes discussed in the text. personalized, image-guided planning: the CPU utilizes intraoperative TRUS to generate a 3D surgical plan based on individual anatomy. This corresponds to the text’s description of a “multidimensional view” and enables the personalized treatment that supports predictable efficacy, especially in larger (50–150 cm^3^) prostates as reported in the WATER studies. Precise, nonthermal robotic resection: The Articulating Armpositions the Handpiece, which delivers a nonthermal, high-velocity saline jet. This core mechanism of “accurate, nonthermal tissue removal”provides the technical basis for minimizing collateral thermal damage, which is fundamental to the potential for preserved sexual (ejaculatory) function noted in clinical trials. Efficient integrated workflow: components such as the console and motorpack support a controlled procedural sequence. This integrated, robotic execution is consistent with the reported efficiency of the method, contributing to the short average resection time of 4 to 5 min. CPU = conformal planning unit.

The AquaBeam Robotic System (PROCEPT BioRobotics, Redwood City, California, USA) is an ultrasound-guided surgical technique that utilizes a nonthermal high-energy water jet to accurately remove obstructive prostate tissue, thereby relieving symptoms.^[31,67,68]^ Its uniqueness lies in the integration of a combination of cystoscope, intraoperative TRUS imaging, and advanced planning software, providing surgeons with a multidimensional view of the surgical area.^[39]^ This allows for personalized treatment planning based on the patient’s prostate anatomy and real-time monitoring of the surgical procedure. Following the ablation, focal bladder neck coagulation (FBNC) may be performed using a standard cystoscope, followed by the insertion of a 3-way catheter for continuous bladder irrigation or gentle traction using a Foley catheter balloon for hemostasis^[30,69]^ (Fig. 9).

The first pivotal trial, the WATER study, is a prospective, double-blinded, multicenter international clinical trial that compared the safety and efficacy of Aquablation and TURP in treating LUTS caused by BPH.^[39]^ A total of 181 male patients were randomly assigned in a 2:1 ratio to receive either Aquablation (116 males) or TURP (65 males). At 6 months, patients treated with Aquablation and TURP both experienced significant improvements in IPSS. Compared to TURP, men treated with Aquablation had a lower ejaculation rate (10% vs 36%, *P* <.0003) and demonstrated more pronounced safety and efficacy benefits in larger prostates (50–80 cm^3^). The WATER-II trial^[70]^ was a prospective, multicenter, single-arm international clinical trial that studied the outcomes of patients with prostate volumes ranging from 80 to 150 cm^3^ after Aquablation treatment. The trial compared the results of 116 subjects from the WATER trial and 101 subjects from the WATER-II trial after receiving Aquablation treatment. The findings showed that IPSS decreased from a baseline of 22.9 and 23.2 to 8.0 and 6.5 at 36 months, respectively. Qmax increased by 11.2 and 9.8 mL/s, indicating significant improvements in both IPSS and Qmax. Within this 36-month period, 98% of patients in the WATER trial and 94% in the WATER-II trial did not require BPH medication, and only 4% and 3% of treated patients, respectively, required surgical intervention.

A study analyzing the 5-year results comparing Aquablation with TURP found that the improvement in IPSS was 15.1 points in the Aquablation group compared to 13.2 points in the TURP group. However, for men with larger prostates (≥50 cm^3^), the Aquablation group had a 3.5 percentage point higher reduction in IPSS at all follow-up visits compared to the TURP group (*P* = .0123).^[20]^ Qmax improved by 125% and 89% from baseline in the Aquablation and TURP groups, respectively. Additionally, the Aquablation group had a 51% lower need for medication or surgical intervention postoperatively compared to the TURP group.

Compared to TURP, Aquablation demonstrates faster resection time, with an average resection time of approximately 4 to 5 minutes.^[25,39]^ The average total procedure time for Aquablation and TURP is similar (33 minutes vs 36 minutes), with an average hospital stay of 1.4 days.^[39]^ The aforementioned studies indicate that for patients with prostate volumes between 30 and 80 cm^3^, Aquablation shows comparable functional outcome improvements to TURP, with shorter surgical and hospitalization times. It also offers advantages such as preserving sexual function, shorter learning curve, and reduced rates of retreatment. Additionally, several studies have reported the potential effectiveness of Aquablation in treating larger prostate volumes (80–150 cm^3^).^[26,39,71]^ Bleeding is the most common serious adverse event associated with Aquablation. However, this risk has been shown to be effectively managed with standardized intraoperative hemostasis, particularly through non‑resective FBNC. A large 2021 study involving over 2000 patients reported that with the adoption of such techniques, the overall transfusion rate can be as low as 0.8%.^[69]^ Earlier data indicated that the transfusion risk was historically higher and correlated with prostate size, and that techniques such as balloon tamponade alone were less effective than when combined with FBNC.^[72]^ Thus, FBNC has now become a key technique for minimizing bleeding complications associated with Aquablation. With the implementation of new hemostatic strategies and increased clinical application, it can be predicted that the incidence of bleeding complications will continue to decrease, further enhancing the status of Aquablation as a treatment option for LUTS symptoms caused by BPH.^[71]^ In conclusion, although the efficacy and safety of Aquablation treatment still require further clinical trials and time for validation, it can be considered as a treatment option for patients with prostate volumes smaller than 80 cm^3^ (conditionally recommended, level C evidence).^[7]^ The EAU2023 guidelines also suggest that Aquablation treatment can be offered as an alternative to TURP for patients with moderate to severe LUTS and prostate volumes between 30 and 80 cm^3^ (weak recommendation; level C evidence).^[22]^

## 9. Conclusion

At present, TURP remains the gold standard surgical treatment for LUTS caused by BPH). However, its inherent limitations – including risks of bleeding, urinary incontinence, RR, ED, and transurethral resection syndrome,^[12–14,73]^ as well as limited efficacy for large-volume prostates (>80 cm^3^) – have driven the rapid development of various MISTs as alternative options.

The 5 MISTs discussed in this review (Rezum, PUL, PAE, iTIND, and Aquablation) are not equivalent in terms of efficacy, and their strength of evidence and clinical positioning vary significantly. Aquablation and PUL possess the highest level of evidence from randomized controlled trials and have received clear recommendations from both AUA2021 and EAU2023 guidelines for specific patient populations (e.g., prostate volume <80 cm^3^), establishing them as rigorously validated alternatives to TURP. Rezum has robust medium- to long-term data and serves as an effective option for preserving ejaculatory function, although its recommendation levels and applicable scopes differ among guidelines. PAE, as a completely noninvasive interventional technique, provides an important option for high-surgical-risk patients, but its efficacy (particularly long-term) requires further validation, and it is currently primarily used within clinical trials or specific circumstances. iTIND, as an emerging technology, shows promising short-term outcomes, but lacks long-term data and has not yet been formally recommended by major guidelines.

This evolution in the treatment landscape signifies a paradigm shift in BPH management – from a singular focus on symptom relief towards evidence-based, individualized selection. The core of decision-making lies in precisely matching the patient’s prostate anatomy, symptom profile, desire for functional preservation (especially sexual function), comorbidities, and personal preferences with the specific evidence base, advantages, and limitations of each MIST.

Looking ahead, more long-term, head-to-head comparative studies are needed to further define the precise role of each MIST within specific patient subgroups. With the continuous enrichment of evidence and ongoing technological advancements, we are poised to offer truly personalized and optimized treatment plans for a broader patient population. Advantages and disadvantages of minimally invasive surgical therapies compared to TURP (authors’ estimation based on the available evidence).

Supplemental digital content “OA Supplemental Content” is available for this article (https://links.lww.com/MD/R531).

## Author contributions

**Conceptualization:** Xintao Zhang, Hongjun Gao.

**Data curation:** Xintao Zhang, Taisheng Liang, Yu Dong.

**Formal analysis:** Xintao Zhang, Taisheng Liang.

**Funding acquisition:** Hongjun Gao.

**Investigation:** Xintao Zhang, Taisheng Liang, Yu Dong.

**Methodology:** Xintao Zhang, Hongjun Gao.

**Project administration:** Hongjun Gao, Xintao Zhang.

**Resources:** Hongjun Gao.

**Supervision:** Hongjun Gao.

**Validation:** Xintao Zhang, Taisheng Liang, Yu Dong, Hongjun Gao.

**Visualization:** Taisheng Liang,Yu Dong.

**Writing – original draft:** Xintao Zhang.

**Writing – review & editing:** Xintao Zhang, Taisheng Liang, Yu Dong, Hongjun Gao.

## Supplementary Material


