# Supplementary material for: Minimally invasive treatments for benign prostatic hyperplasia: A narrative review
Source: Medicine (Baltimore). 2026 Mar 20;105(12):e47951. doi: 10.1097/MD.0000000000047951 (PMC13008176; doi:10.1097/MD.0000000000047951)
Supplement: Supplementary file 1 [file medi-105-e47951-s001.docx]

11

**Scale** **for** **the** **Assessment** **of** **Narrative** **Review** **Articles-SANRA**

Please rate the quality of the marrative review article in question, using categories 0-2 on the following scale. For each aspect of quality, please choose the option which best fits your evaluation, using categories 0 and 2 freely to imply general low and high quality.These are not intended to imply the worst or best imaginable quality.

**1) Justification of the article's importance for the readership**

The importance is not justified. 0

The importance is alluded to, but not explicitly justified. 1

The importance is explicitly justified 2

**2) Statement of concrete aims or formulation of questions**

No aims or questions are formulated. 0

Aims are formulated generally but not concretely or in terms of clear questions. 1

One or more concrete aims or questions are formulated. 2

**3) Description of the literature search**

The search strategy is not presented. 0

The literature search is described briefly. 1

The literature search is described in detail, including search terms and inclusion criteria. 2

**4) Referencing**

Key statements are not supported by references. 0

The referenceing of key statements is inconsister. 1

Key statements are supported by references. 2

**5) Scientific reasoning**

(e. g, incorporation of approprinte evidence, such as RCTs in clinical medicine)

The article's point is not based on appropriate arguments. 0

Appropriate evidence is introduced selectively. 1

Appropriate evidence is generally present. 2

**6) Appropriate presentation of data**

Data are presented inadequately. 0

Data are often not presented in the most appropriate way. 1

Relevant outcome data are generally presented appropriately. 2

**Sumscore**

1

2

2

2

2

2
